# Untargeted metabolomics reveals the alteration of metabolites during the stewing process of Lueyang black-bone chicken meat

**DOI:** 10.3389/fnut.2024.1479607

**Published:** 2024-11-27

**Authors:** Ling Wang, Guojin Li, Jie Gao, Jia Cheng, Zhengnan Yuan, Hongzhao Lu, Wenxian Zeng, Tao Zhang

**Affiliations:** ^1^School of Biological Science and Engineering, Shaanxi University of Technology, Hanzhong, China; ^2^Shaanxi University Engineering Research Center of Quality Improvement and Safety Control of Qinba Special Meat Products, Hanzhong, China; ^3^Qinba Mountain Area Collaborative Innovation Center of Bioresources Comprehensive Development, Shaanxi University of Technology, Hanzhong, China; ^4^Qinba State Key Laboratory of Biological Resources and Ecological Environment, Shaanxi University of Technology, Hanzhong, China; ^5^Shaanxi Baiweiyuan Network Technology Company, Hanzhong, China

**Keywords:** black-bone chicken, stewing, chicken soup, untargeted metabolomics, unsaturated lipids

## Abstract

**Introduction:**

Black-bone chicken meat is rich in nutritional substances and bioactive compounds. Stewing is a traditional and healthy cooking style for black-bone chicken meat. However, the alteration of metabolites in chicken meat during stewing is still unknown.

**Methods:**

A comprehensive analysis of Lueyang black-bone chicken meat metabolites was performed in fresh chicken meat (FM), short-term heat-pretreated meat (PM), fully cooked meat (CM) and chicken soup (CS) via untargeted metabolomics.

**Results:**

By comparison, 200, 992 and 891 significantly differentially metabolites (DMs) were identified in the PM vs. FM, CM vs. FM and CS vs. FM comparisons, respectively. These DMs mainly included amino acids, peptides, carbohydrates and lipids. During the heating process, the abundances of Ser, Ala, Tyr, niacinamide, galactose, guanosine 3′-monophosphate and inosine 5′-monophosphate in chicken meat significantly decreased and were partially dissolved in the soup. Due to the hydrolysis of phospholipids, the relative contents of unsaturated lipids, especially a range of lysophosphatidylcholines, lysophosphatidylethanolamines, arachidonic acid and derivatives, increased in fully cooked meat.

**Discussion:**

Pretreatment had little impact on the changes in metabolites in chicken meat. During stewing, the dissolved amino acids, carbohydrates and nucleic acids could enhance the taste quality of chicken soup, and the high abundance of unsaturated lipids could promote the nutritional quality of black-bone chicken meat. In summary, these data provide helpful information for nutritional quality studies on the metabolite profiles of black-bone chicken meat.

## 1 Introduction

In traditional Chinese dietary culture, black-bone chickens are specific chicken breeds whose meat and bones are rich in nutritional value and medicinal properties (1). As a nutritious food for humans, black-bone chicken meat contains a variety of amino acids, including 8 essential amino acids. Special amino acids, including but not limited to Glu, Asp, Gly, Ala and Leu, in black-bone chicken meat are antioxidants with reducing power and radical scavenging capacity (2). Additionally, a variety of characteristic peptides in black-bone chickens have immunomodulatory effects by significantly enhancing the proliferation of T and B lymphocytes and the phagocytosis of macrophages (3). Compared with red meat, black-bone chicken meat contains many unsaturated fatty acids (especially linoleic acid and linolenic acid) and less cholesterol, and these unsaturated fatty acids are necessary for the normal growth and development of humans and are even beneficial for the prevention of cardiovascular diseases (4). In addition, melanin pigmentation in various tissues, such as bone, skin, and muscle, is a distinct feature of black-bone chickens. Melanin has a wide range of biochemical activities, such as antioxidation, anti-inflammatory and immunoregulatory functions (5, 6). It is important to mention that black-bone chicken meat has certain medicinal value for curing headache, hepatitis, asthma and other heart diseases (7). Lueyang black-bone chickens are local specific broilers raised in the Chinese Qinling Mountains. The meat of Lueyang black-bone chickens is rich in polypeptides, amino acids, unsaturated fatty acids, vitamins, and melanin and has extremely high nutritional and medicinal value (8).

To ensure the preservation of nutrients in black-bone chicken meat, stewing is traditionally the preferred cooking style. Before stewing, the chicken meat undergoes pretreatment, also referred to as short-term heating, to eliminate blood, diminish the unusual smell of the raw chicken meat. During the heating process, some of the nutrients in the meat are changed and small amounts of substance will be extracted and released into the soup. According to previous reports, the heating process changes the microstructure of muscle fibers and the physicochemical properties of meat through destruction of cell membranes, coagulation and denaturation of proteins, and lipid degradation (9, 10). Furthermore, various substances in chickens, such as amino acids and derivatives, nucleotides, sugars and peptides, are released into water, which is beneficial to the nutritional and organoleptic qualities of chicken soup (11, 12). Previous studies have focused on the effects of different treatments on the metabolites and flavor substances of chicken soup (13, 14). However, as a traditional Chinese cooking technique, the precise impact of pretreatment on chicken remains unclear. Additionally, the alterations in metabolites during the cooking process of black-bone chicken meat are also not well understood.

Currently, metabolomics serves as the primary approach to study the overall small molecular substances in meat (15, 16). Metabolomics encompasses the qualitative and quantitative analysis of all small molecule metabolites. Liquid chromatography-mass spectrometry (LC-MS) is predominantly utilized for the analysis of small molecule metabolites, while gas chromatography-mass spectrometry (GC-MS) is employed for the analysis of volatile organic compounds. Zhang et al. (17) demonstrated the influence of glycerophospholipids, peptides, and flavonoids on the flavor profile and beneficial properties of Beijing-you chicken breast meat via LC-MS. The effect of ultrasound-assisted stewing on the aroma profile of chicken soup was investigated via HS-SPME-GC-MS by Qi et al. (18). In this investigation, we utilized LC-MS to identify overall metabolites present in raw, pre-treated, fully cooked black-bone chicken meat, and chicken soup. Our aim was to systematically explore the changes and liberation of nutritional components within the meat during the stewing. The insights gained from this study are poised to enhance our comprehension of the nutritional profile of black-bone chicken meat and soup.

## 2 Materials and methods

### 2.1 Ethics approval statement

All animal experimental procedures were approved and conducted in accordance with the guidelines of the Institutional Animal Care and Use Committee of Shaanxi University of Technology (SLGQD/09/2017).

### 2.2 Sample collection and treatment

Six-month-old Lueyang black-bone chickens were euthanized using carbon dioxide anesthesia and conventional neck cutting. The breast and leg (thigh) muscles were isolated and cut into small pieces (1.0 cm width × 1 cm thickness × 1.5 cm length). Breast and leg muscles (100 g) were mixed equally to obtain fresh meat (FM). Before cooking, the fresh meat was treated in boiling water for 3 min and taken as pre-treated meat (PM). Subsequently, the pre-treated meat was added to small tanks supplemented with clean water and salt. Each tank contained approximately 60 g of pretreated meat, 1 g of salt and 200 mL of clean water. Then, the tanks were sealed under vacuum conditions, heated under pressure at 100°C for 15 min and 121°C for 10 min, and cooled immediately with cold water. Thus, the chicken soup was prepared. Fully cooked meat (CM) in the soup and soup only (CS) were collected as metabolic samples, with six biological replicates for each group.

### 2.3 LC-MS analysis of metabolites

Fresh meat (FM), pre-treated meat (PM), cooked meat (CM) and chicken soup (CS) were collected for untargeted metabolomics analysis via LC-MS. Metabolite extraction and quality control were performed by the standard sample preparation process of Majorbio Bio-Pharm Technology Co., Ltd. (Shanghai, China) (19). Briefly, the solid sample (50 mg) was placed in a 2 mL tube with 400 μL of extraction solution [methanol: water = 4:1 (v:v)] containing 0.02 mg/mL of internal standard (L-2-chlorophenylalanine) for metabolite extraction. Then, a 6 mm diameter grinding bead was added to the tube. Samples were subsequently minced by the Wonbio-96c (Shanghai Wanbo Biotechnology Co., Ltd.) frozen tissue grinder at −10°C for 6 min (50 Hz), followed by low-temperature ultrasonic extraction at 5°C for 30 min (40 kHz). The samples were incubated at −20°C for 30 min to facilitate metabolite extraction, and then centrifuged at a speed of 13,000 rpm for 15 min at 4°C. The supernatant was transferred to the injection vial for LC-MS analysis.

The samples were analyzed using LC-MS on a Thermo UHPLC-Q Exactive HF-X system by Agilent Technologies. The chromatography column was an ACQUITY UPLC HSST3 (100 mm × 2.1 mm i.d., 1.8 1; Waters, United States) at Majorbio Bio-Pharm Technology Co., Ltd. The mobile phase A consisted of 95% water and 5% acetonitrile, including 0.1% formic acid. In contrast, mobile phase B was a mixture of 47.5% acetonitrile, 47.5% isopropyl alcohol, and 5% water, with the water component also containing 0.1% formic acid. The sample injection volume was 3 μL, and the flow rate was set to 0.40 mL/min. The column temperature was maintained at 40°C. The mass spectrometric data were acquired utilizing a Thermo UHPLC-Q Exactive HF-X Mass Spectrometer, which was outfitted with an electrospray ionization (ESI) source. The optimal parameters were set as follows: the ion source temperature was set to 425°C, and the capillary temperature was set to 325°C. The sheath gas flow rate was set to 50 arb, while the auxiliary gas flow rate was maintained at 13 arb. The ion-spray voltage floating (ISVF) was established at +3,500 V for positive mode and −3,500 V for negative mode, respectively. The normalized collision energy was set to 20–40–60 V rolling. Full MS resolution was 60,000, and MS/MS resolution was 7,500. The positive and negative ion scanning modes were employed to gather signals. Data was acquired using the data-dependent acquisition (DDA) method. The detection was carried out over a mass range of 70–1,050 m/z. The acquired raw MS files underwent processing using Chroma TOF software (version 3.30, Leco Co., CA) and MATLAB 7.0. Quality control, missing value imputation, and data normalization were performed in accordance with the Majorbio standard protocol.

### 2.4 Metabolic data analysis

LC-MS raw data were imported into Progenesis QI (Waters Corporation, Milford, United States) software for preliminary processing, and finally obtained a three-dimensional data matrix including sample information, metabolite name and mass spectral response intensity. The metabolites were identified by the HMDB,^1^ Metlin,^2^ Majorbio Database and other databases. Subsequently, the data matrix obtained was uploaded to the Majorbio cloud platform^3^ for data analysis. The R package “ropls” (Version 1.6.2) was used to perform principal component analysis (PCA), partial least squares discriminant analysis (PLS-DA) and orthogonal partial least squares discriminant analysis (OPLS-DA). By comparison, metabolites with variable importance in projection (VIP) >1 and adjusted *p* < 0.05 were considered significantly differentially metabolites (DMs). Subsequently, differential metabolites were mapped into their biochemical pathways through metabolic enrichment and pathway analysis based on KEGG database.^4^ Python packages “scipy” (Version 1.0.0)^5^ was used to perform enrichment analysis and metabolite cluster analysis. Time-series expression trend analysis were implemented in the Short Time-series Expression Miner (STEM) software (Version 1.3.11). Key differential metabolites in four groups were screened and dynamic heatmaps were generated by the OmicShare website tool.^6^

## 3 Results and discussion

### 3.1 Quality control of metabolomics from meat and soup

Metabolomic analysis based on LC-MS detection technology was adopted to reveal metabolite alterations during the processing of chicken meat. Heatmaps of sample correlation were generated to evaluate metabolic data reliability in the positive (Supplementary Figure S1A) and negative (Supplementary Figure S1B) ion modes. There was a high similarity in metabolic composition and abundance between the FM and PM samples. Moreover, the metabolic composition and abundance of the FM, CM and CS samples were highly different.

**Figure 1:**
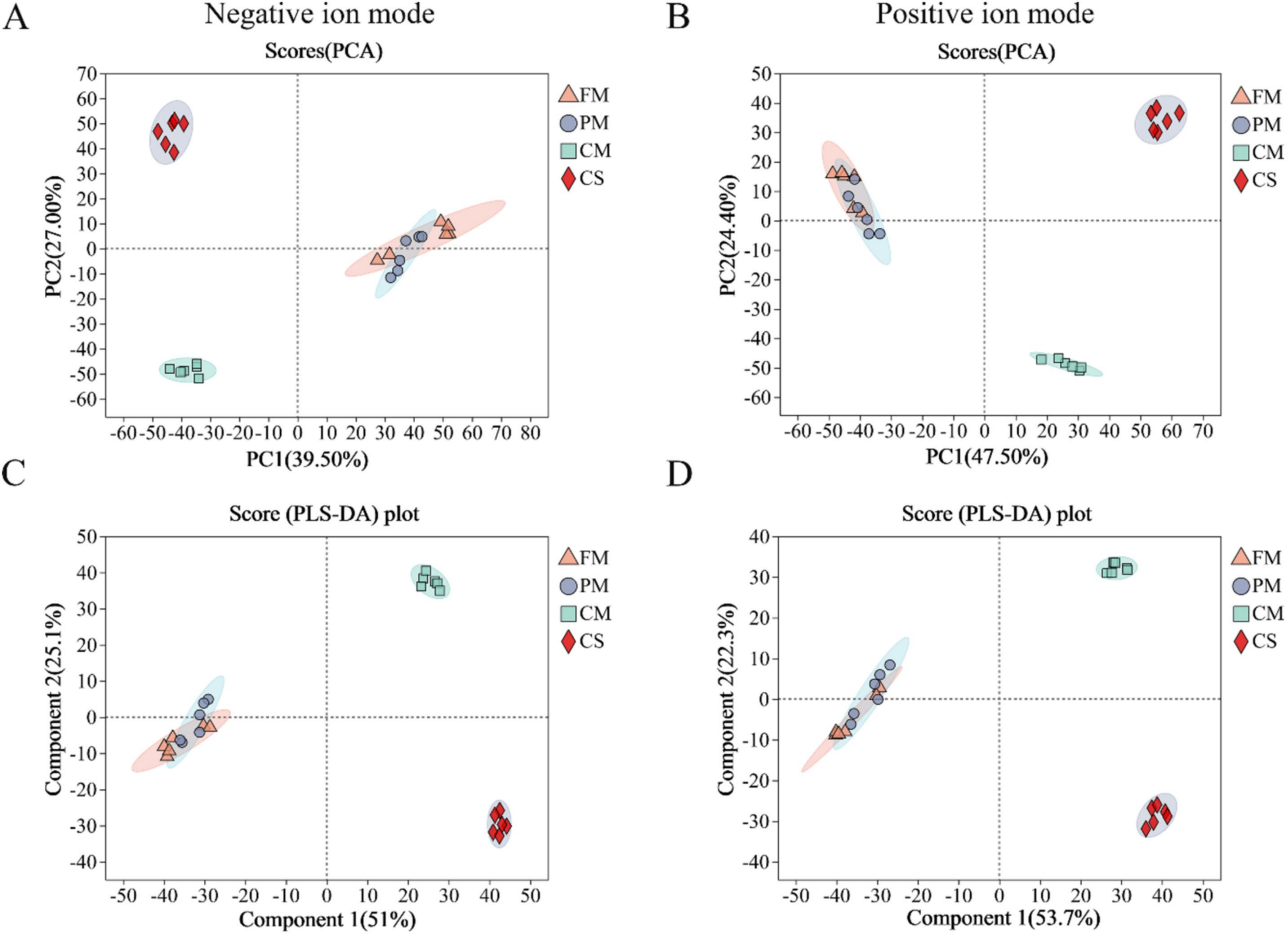
Metabolite profiling analysis of the chicken samples from the four groups by principal component analysis (PCA) and partial least squares discriminant analysis (PLS-DA). PCA of samples under negative mode **(A)** and positive mode **(B)**. PLS-DA analysis of samples in negative mode **(C)** and positive mode **(D)**. FM, fresh meat; PM, pre-treated meat; CM, cooked meat; CS, chicken soup.

Under both negative and positive ion modes, PCA and PLS-DA were performed to further assess the quality of the metabolomics data (Figure 1). Based on principal components 1 and 2, the PCA and PLS-DA score plots demonstrated good repeatability of the experiment, but there was cross-overlap between the FM and PM samples in both modes. In addition, the samples from the CM and CS groups were significantly different among the four groups. All samples were within the 95% confidence interval. These results indicated that short-term heat treatment had little effect on chicken metabolite alterations. However, changes in chicken metabolites were greatly affected during long-term heating.

### 3.2 OPLS-DA analysis of changes in meat metabolites during stewing

**Figure 2:**
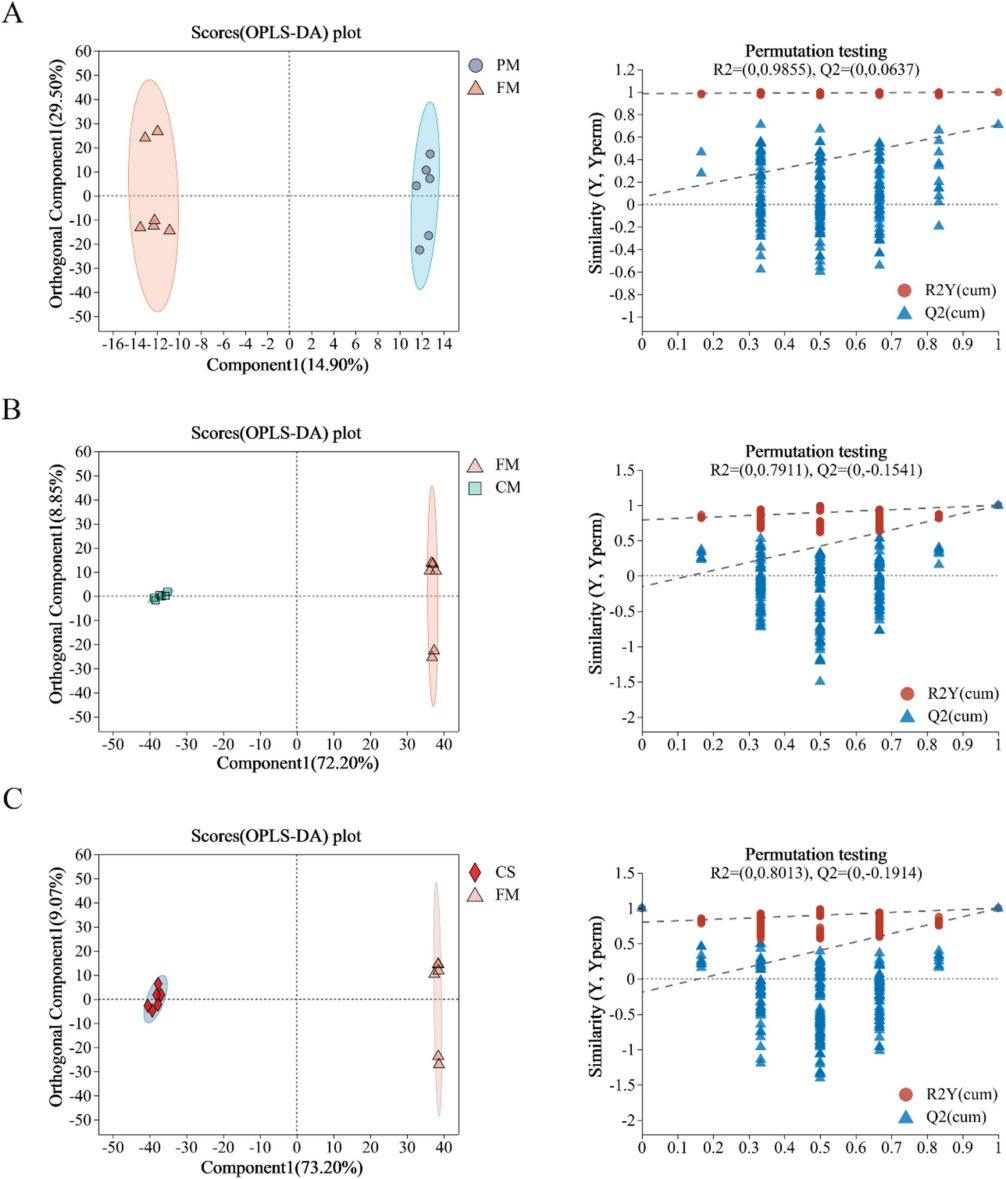
Orthogonal partial least square discriminant analysis (OPLS-DA) and permutation test diagrams of metabolites between PM and FM **(A)**; FM and CM **(B)**; and CS and FM **(C)**.

To further investigate the metabolite changes in chicken meat before and after treatment, OPLS-DA was used to perform pairwise comparisons of data between the PM and FM (Figure 2A), CM and FM (Figure 2B), and CS and FM (Figure 2C) groups. OPLS-DA score plots and 200 permutation tests supported that the established model was reliable and predictable. The permutation plot of the PM and FM data showed distinct overfitting (Figure 2A), but OPLS-DA revealed a significant separation between the two groups. These data suggested that the metabolites of chicken meat before and after pretreatment were quite similar. The explained variation value and the predictive capability between CM and FM were R2X = 0.811, R2Y = 0.999, and Q2 = 0.994 (Figure 2B), and R2X = 0.822, R2Y = 0.999, and Q2 = 0.997 for comparison between CS and FM (Figure 2C), which indicated the reliability of the models. In brief, these data suggested that the levels of metabolites in Lueyang black-bone chicken muscle changed significantly during stewing.

### 3.3 Screening and classification analysis of differentially abundant metabolites

**Figure 3:**
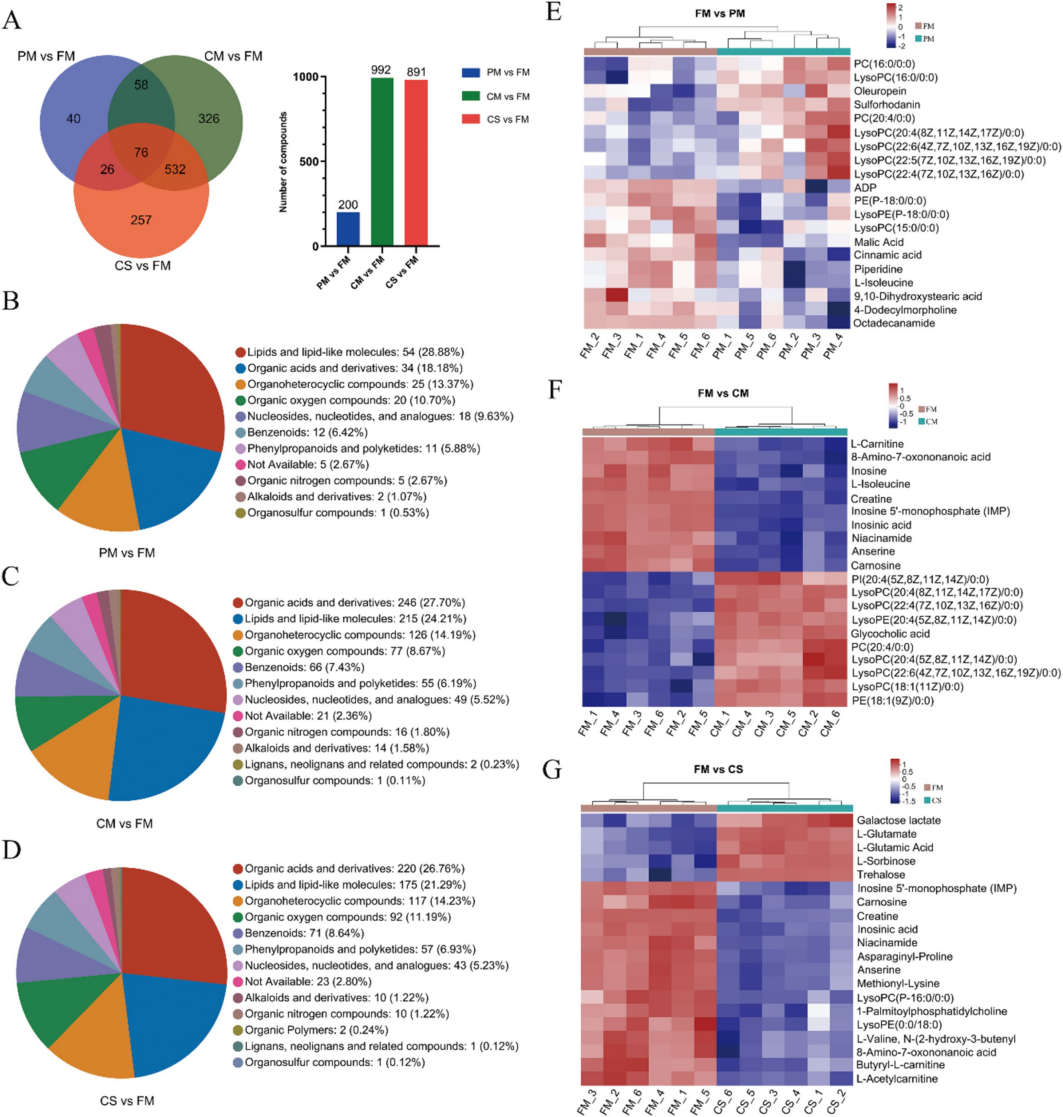
Screening and classification analysis of DMs. **(A)** The overlap of DMs among the three comparisons via a Venn diagram. Chemical compound classifications of DMs were used between the PM and FM **(B)**, CM and FM **(C)**, and CS and FM **(D)** groups. Heatmaps of the top 20 differentially abundant metabolites between the FM and PM **(E)**, FM and CM **(F)**, and FM and CS **(G)** groups.

Based on the cutoffs of VIP >1 and *p* < 0.05 for differentially abundant metabolite screening, a total of 1,315 metabolites were screened from muscles under positive and negative ion modes. We identified 76 DMs that were shared among the three comparisons. Moreover, 200 DMs (114 and 86 DMs in the positive and negative modes, respectively), 992 DMs (603 and 389 DMs in the positive and negative modes, respectively) and 891 DMs (553 and 338 DMs in the positive and negative modes, respectively) were identified in PM vs. FM, CM vs. FM, and CS vs. FM, respectively (Figure 3A). The DMs were clustered into different categories, including lipids and lipid-like molecules, organic acids and derivatives, and organoheterocyclic compounds (Figures 3B–D).

To visualize the changes in metabolites during the process, heatmap analyses were performed for the top 20 DMs in the three comparison groups. Compared with metalites in the FM group, oleuropeina, sulforhodanin and a series of unsaturated lysophosphatidylcholines (LysoPCs), including LysoPC (20:4), LysoPC (22:6), LysoPC (22:5), and LysoPC (22:4), were upregulated in the PM group. Moreover, lysophosphatidylethanolamine [LysoPE (P-18:0)], LysoPC (15:0), malic acid, cinnamic acid, L-isoleucine, 9,10-dihydroxystearic acid, 4-dodecylmorpholine, and octadecanamide were downregulated (Figure 3E). These results suggested that short-term heating leads to the accumulation of unsaturated LysoPCs, while the contents of saturated LysoPE (P-18:0), LysoPC (15:0), L-isoleucine and octadecanamide decreased. Furthermore, comparing DMs between the FM and CM groups, 5 types of unsaturated LysoPC, glycocholic acid and phosphatidylinositol [PI (20:4)] were upregulated in CM samples, while inosine, L-isoleucine, niacinamide and carnosine were downregulated (Figure 3F). Metabolites in chicken soup without meat were extracted from chicken meat during cooking. Generally, fewer metabolites were detected in chicken soup than in fresh chicken meat, except for the metabolites that changed during cooking. In comparison, among the top 20 metabolites, the concentrations of 15 DMs, such as inosine 5′-monophosphate (IMP), carnosine, creatine and niacinamide, were reduced in the chicken soup sample. However, the levels of galactose lactate, L-glutamate, L-glutamic acid, L-sorbinose and trehalose increased in the soup (Figure 3G). These results indicated that some water-soluble amino acids and sugars were easily released and dissolved in the soup, increasing the nutrient content of the soup.

### 3.4 DMs enriched KEGG classification and analysis

**Figure 4:**
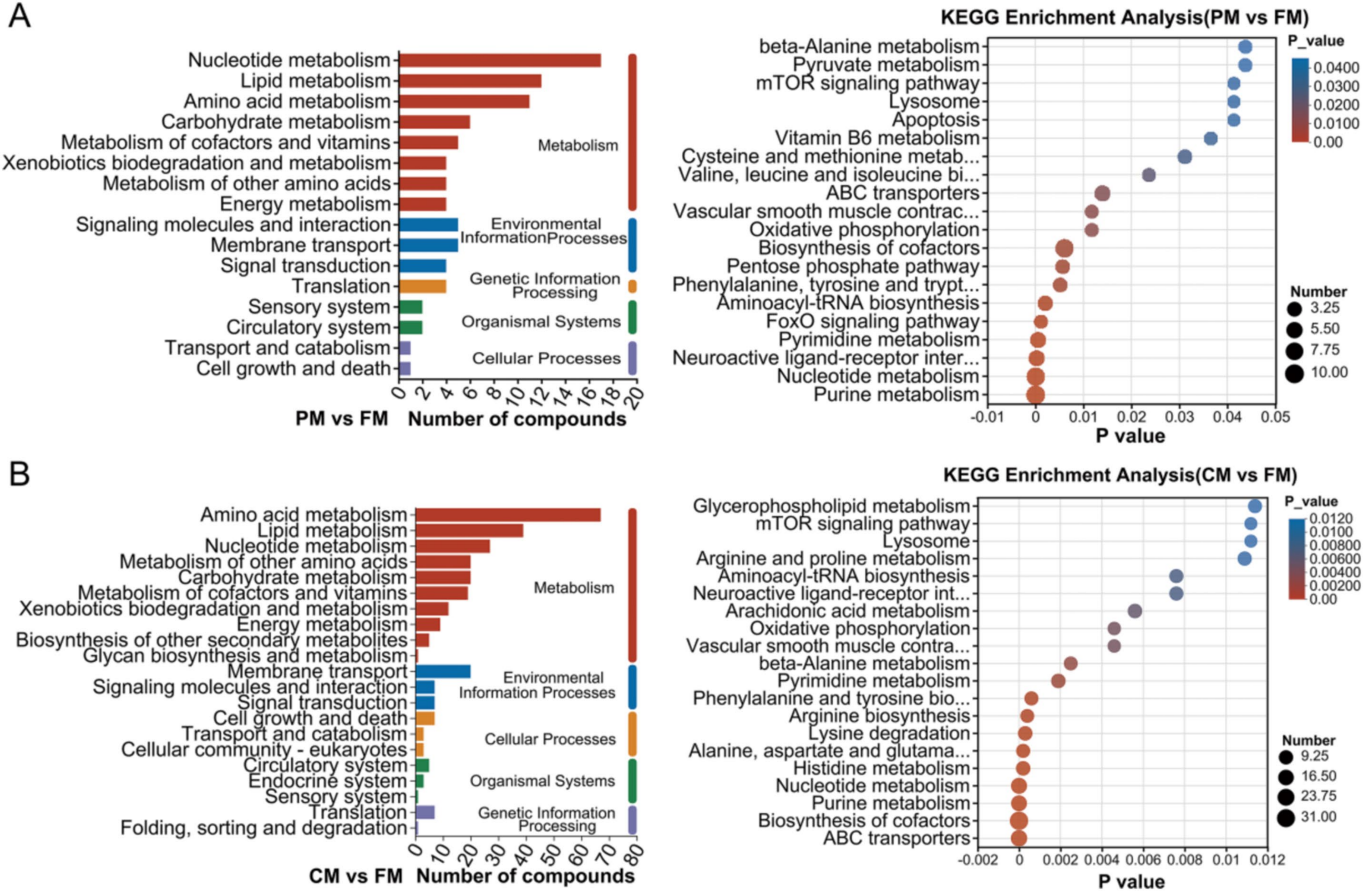
KEGG enrichment analysis of DMs between PM and FM **(A)**, CM and FM **(B)**.

Based on the KEGG database, a total of 16 and 21 KEGG pathways were identified in the PM vs. FM and CM vs. FM. These enriched pathways were mainly classified as metabolism, environmental information processes, cellular processes, organismal systems and genetic information processing (Figure 4). Many more compounds are involved in metabolism, especially nucleotide metabolism, lipid metabolism and amino acid metabolism. DMs between PM and FM were mainly enriched in purine metabolism (12 DMs), nucleotide metabolism (10 DMs) and biosynthesis of cofactors (10 DMs) (Figure 4A). The DMs between CM and FM were predominantly involved in ABC transporters (20 DMs), biosynthesis of cofactors (34 DMs), and purine metabolism (18 DMs) (Figure 4B). These data indicated that compounds predominantly related to nucleotide metabolism, lipid metabolism and amino acid metabolism, greatly changed during thermal processing. Nucleotide metabolism, lipid metabolism and amino acid metabolism are intricately linked to the formation of the flavor substances. These metabolic pathways play a crucial role in shaping the taste of food by producing distinct flavor compounds, including aldehydes, ketones, alcohols, and acids (20).

### 3.5 Analysis of DM alteration trends during stewing

The total number of DMs among the four groups was collected for short time-series expression miner analysis. Based on 1,352 DMs, 4 significant clusters were identified in 10 trend analysis charts (Supplementary Figure S2).

**Figure 5:**
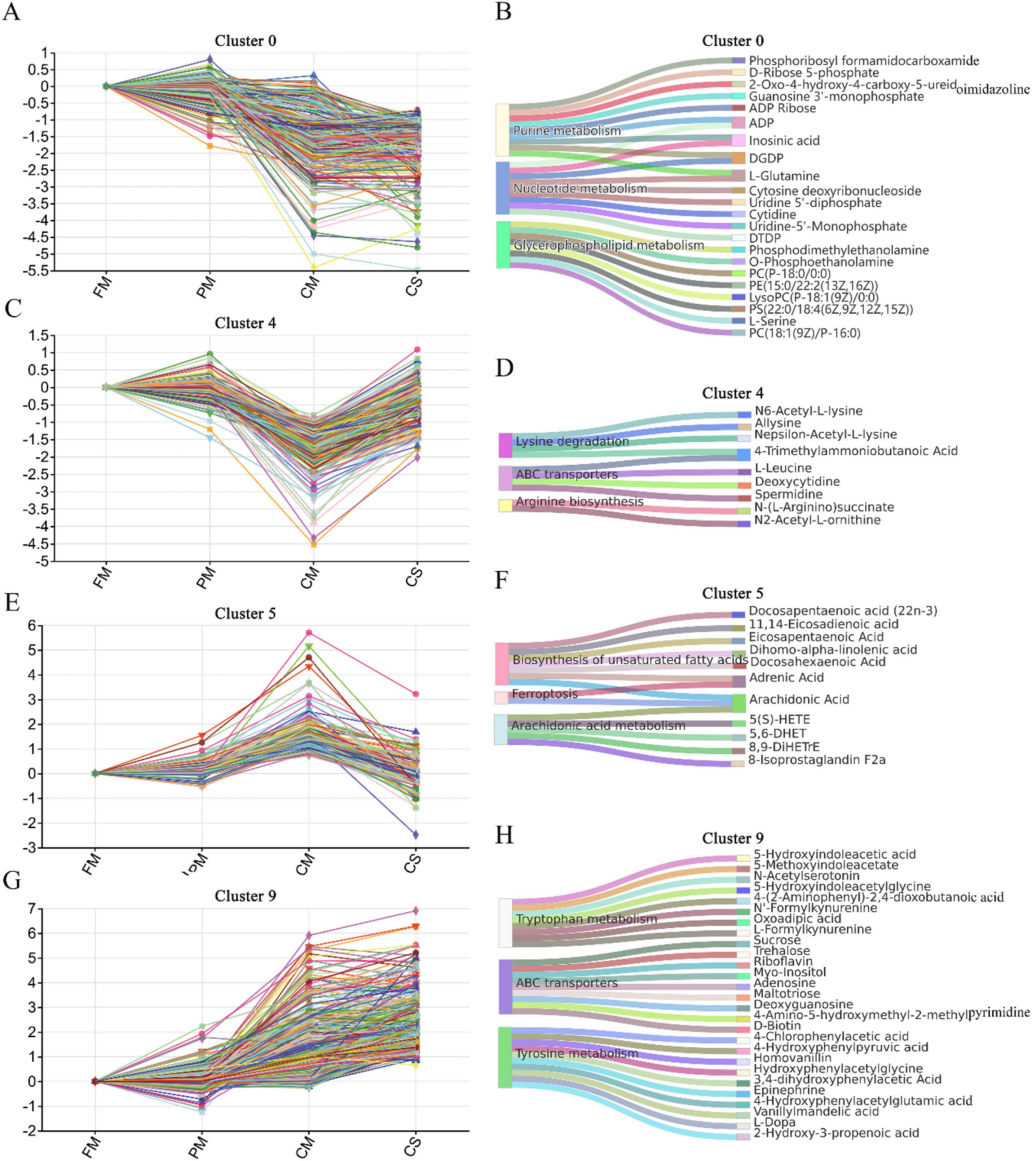
Significant clusters of DM expression trends during the heating process. Expression trends of DMs and the top three KEGG pathways enriched in cluster 0 **(A,B)**, cluster 4 **(C,D)**, cluster 5 **(E,F)** and cluster 9 **(G,H)**.

In cluster #0, 301 DMs gradually degraded during the heating process, and their concentrations significantly decreased in the CM and CS groups (Figure 5A). These DMs were mainly enriched in nucleotide metabolism (9 DMs), glycerophospholipid metabolism (8 DMs), and purine metabolism (9 DMs) (Figure 5B). The main compounds involved in nucleotide metabolism were inosinic acid, uridine-5′-monophosphate (UMP), uridine 5′-diphosphate (UDP), and cytidine. The main compounds involved in purine metabolism were guanosine 3′-monophosphate (GMP), ADP, and D-ribose 5-phosphate. These nucleotides may be degraded during the heating process in chicken meat and released into the soup. In addition, phosphatidylcholine [PC (18:1(9Z)/P-16:0)], phosphatidylethanolamine [PE (15:0/22:2(13Z, 16Z))], phosphatidylserine [PS (22:0/18:4(6Z, 9Z, 12Z, 15Z))] and other unsaturated phospholipids participate in glycerophospholipid metabolism and are gradually degraded during the heating process. The main reason for the loss of these unsaturated phospholipids is lipid oxidation (21).

In cluster #4, 214 DMs, which were obviously increased in the CS groups compared with those in the CM groups, were mainly enriched in amino acid metabolism, such as lysine degradation (4 DMs), ABC transporters (4 DMs) and arginine biosynthesis (2 DMs) (Figures 5C,D). N6-acetyl-L-lysine, ε-acetyl-L-lysine, 4-trimethylammoniobutanoic acid and allysine were mainly enriched in lysine degradation. Deoxycytidine, L-leucine, spermidine and 4-trimethylammoniobutanoic acid are related to ABC transporters. N-(L-Arginino) succinate and N2-acetyl-L-ornithine were enriched in arginine biosynthesis. These data suggested that various substances, such as lysine, leucine and ornithine, were extracted from the meat and dissolved in the soup during stewing.

In cluster #5, 121 DMs showed higher concentrations in the CM samples and lower concentrations in the CS samples (Figure 5E). These DMs were significantly enriched in the biosynthesis of unsaturated fatty acids (7 DMs) and arachidonic acid metabolism (5 DMs) (Figure 5F). The contents of unsaturated fatty acids, such as arachidonic acid, dihomo alpha-linolenic acid, adrenic acid, and docosahexaenoic acid 5(S)-HETE, were relatively high in the CM samples. These data indicated that the heating process leads to the formation of unsaturated fatty acids. Because most unsaturated fatty acids are insoluble in water, these substances accumulate in cooked black-bone chicken meat during stewing.

In cluster #9, 451 DMs continuously increased from fresh meat to soup (Figure 5G). These DMs were mainly enriched in tyrosine metabolism (10 DMs), tryptophan metabolism (8 DMs) and ABC transporters (8 DMs) (Figure 5H). Hydroxyphenylacetylglycine, 4-chlorophenylacetic acid and 4-hydroxyphenylpyruvic acid participate in tyrosine metabolism. Formylkynurenine, 5-hydroxyindoleacetic acid and acetylserotonin are involved in tryptophan metabolism. Trehalose, adenosine, and sucrose participate in ABC transporters. These results indicated that these substances accumulated during the heating process in the meat and largely dissolved in the chicken soup.

### 3.6 Analysis of key compound profiles

**Figure 6:**
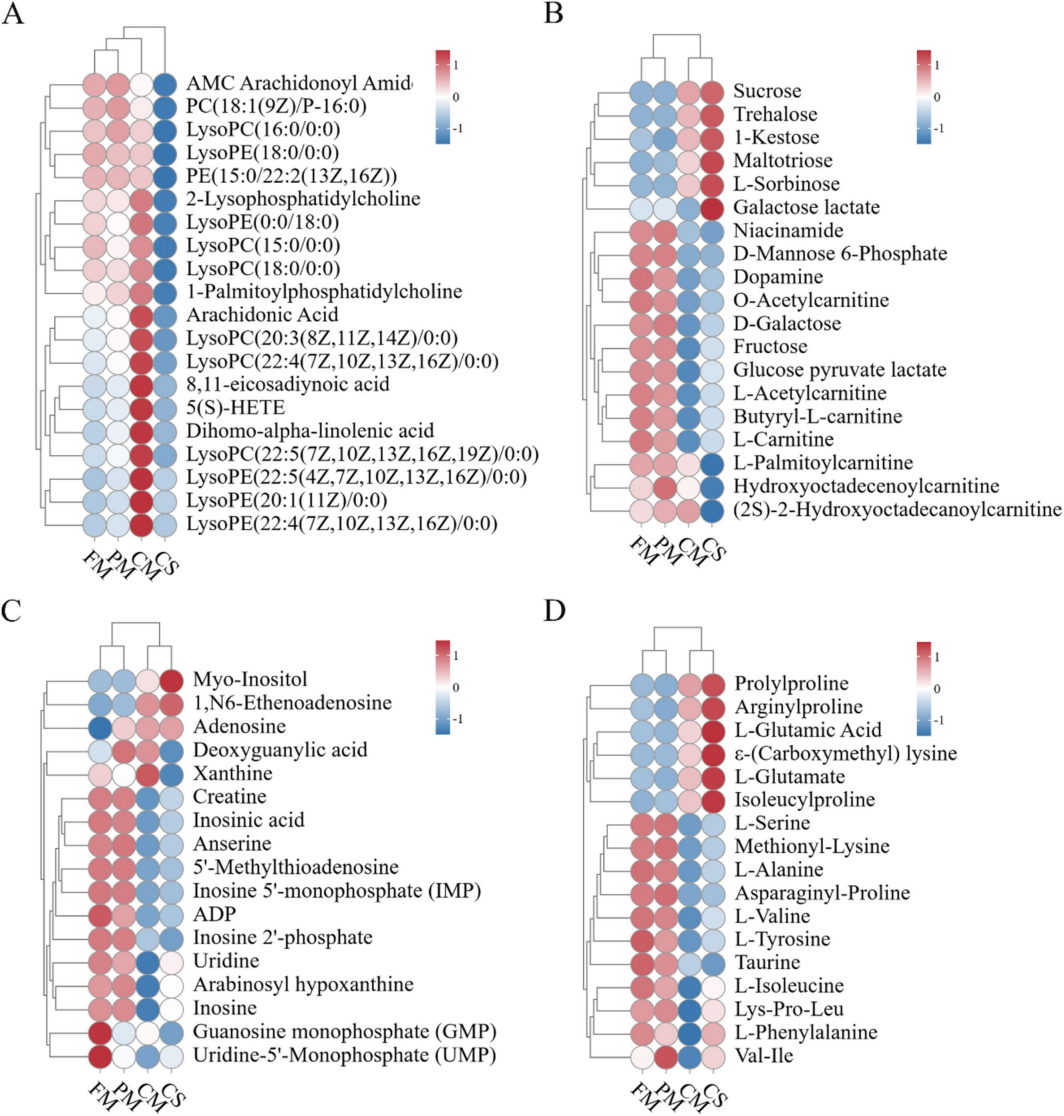
Heatmap analysis of key compounds. **(A)** Lipids. **(B)** Carbohydrates. **(C)** Nucleic acids. **(D)** Amino acids and peptides.

To further investigate the changes in vital metabolites in chicken meat and soup during the stewing process, the changes in lipids, carbohydrates, nucleic acids, amino acids and peptides were analyzed (Figure 6). These compounds are vital nutrients for humans. In particular, amino acids and nucleic acids largely contribute to the taste and aroma of chicken meat and soup (11, 12).

For lipids, a large series of phospholipids (PC, PE, PI, and PS) were identified from chicken meat during the stewing process. Hydrolysis of phospholipids results in the formation of lysophospholipid (LPL) and fatty acids by the intracellular phospholipases A1 (PLA1) and phospholipase A2 (PLA2) (22). We found that the contents of 6 LysoPCs and 5 Lysophosphatidylethanolamines (LysoPEs) increased during heating and were relatively highly abundant in the CM sample (Figure 6A). These data were consistent with those in a previous study, which illustrated that heating at 70°C and 80°C obviously increased the LysoPC content in chicken meat (21). Additionally, Luo et al. (23) reported that the number of LPL molecules in chicken egg yolk increased under high heat treatment. Besides, chicken intestinal secretory group phospholipase A2 maintained approximately 60% of its activity after 15 min of incubation at 60°C (22). Another previous study reported that recombinant PLA2 from wheat maintained 45% of its activity after treatment at 100°C for 5 min (24). As the heating temperature increased, the production of LysoPC was enhanced by phospholipase-mediated hydrolysis of phospholipids. LysoPC was found to have anti-inflammatory, anti-hemostatic, and cytotoxic effects (25). Additionally, fatty acids are simultaneously released from the hydrolysis of phospholipids (26). In our study, the contents of arachidonic acid, 8,11-eicosadiynoic acid, 5(S)-HETE, and dihomo-alpha-linolenic acid increased during the stewing process (Figure 6A). Previous studies have shown that arachidonic acid can improve the taste of chicken meat and plays essential roles in human immune, cardiovascular, and nervous system development (27, 28). As a lipoxygenase metabolite of arachidonic acid, 5(S)-HETE impacts DNA synthesis in human microvascular endothelial cells (29).

For carbohydrates, the content of maltotriose increased from FM to CM. Due to its heat resistance, maltotriose is stable at high temperatures and is predominantly responsible for its sweet taste (Figure 6B). Li et al. (30) reported that the content of maltotriose dramatically increased in cooked donkey meat. Compared with those in meat before stewing, the levels of 17 carbohydrates, including niacinamide, fructose and D-galactose, decreased in CM. Niacinamide is a well-known antioxidant that is not stable at high temperature. Thus, niacinamide in chicken meat was degraded during heating. Wang et al. (31) reported a substantial reduction in D-fructose in chicken egg yolks via Maillard reactions during long-term heating. Furthermore, L-fructose and D-galactose may enhance meat flavor and increase the water holding capacity by inducing proteoglycan and myosin solubilization (32). Here, we also found a decrease in muscle carnitine, which was consistent with previous findings (33, 34). Carnitine, a water-soluble nutrient, can contribute to the production of energy and transport long-chain fatty acids to mitochondria for β-oxidation (35, 36).

For nucleic acids, the relative contents of myo-inositol, adenosine, and 1,N6-ethenoadenosine were greater in both CM than in FM and PM (Figure 6C). The increase in adenosine and 1,N6-ethenoadenosine may be largely due to the degradation of ATP during heating. However, the abundances of anserine, carnosine and creatine were substantially lower in CM than in FM. Anserine, carnosine and creatine are bioactive compounds that are present in high concentrations in meat and readily degrade during the heating process (37). Anserine and carnosine generate bitterness (38). The reduction in anserine and carnosine in this study alleviated the increase in the bitterness of chicken meat. Additionally, inosine and inosinic acid are dietary nucleotides closely associated with meat flavor formation. IMP and GMP, which are umami compounds, strongly contribute to meat flavor via synergistic effects with L-glutamate (39, 40). Heat treatment can decrease the IMP content via degradation or reactions with other compounds (41).

The levels of 6 amino acid and peptide metabolites, including L-glutamate and N-ε-(carboxymethyl) lysine, were relatively high in the CM (Figure 6D). The abundances of 11 compounds, including L-serine, L-alanine and methionyl-lysine, were substantially reduced in the CM. According to Maillard reactions, lysine and D-fructose are readily converted into N-ε-carboxymethyl-lysine or N-ε-carboxyethyl-lysine. Wang et al. (31) reported that L-lysine was significantly reduced in chicken egg yolks during heat treatment. In the present study, we found that after heating, the amount of methionyl-lysine decreased but the amount of N-ε-(carboxymethyl) lysine increased in CM. Furthermore, amino acids and their derivatives are crucial flavors and tastes for meat and soup. Previous studies have demonstrated that amino acids impart sweet (Gly, Ala, Ser, Thr, Pro, Hy-pro), sour (Phe, Tyr, Ala), bitter (His, Arg, Ile, Leu, Lys, Phe, Val, Tyr), and umami (Asp and Glu) tastes to meat (42, 43). In the present study, glutamine and glutamic acid were relatively high in CM and enhanced the sweet and umami taste. The contents of Ala, Val, Ile, and Tyr, which impart bitter taste, decreased in chicken meat after heat treatment. These data indicated that stewing could reduce the amount of bitter taste and enhance the amount of sweet and umami taste in Lueyang black-bone chicken meat.

Chicken soup is a simple but nutritious food, frequently cherished by people for its distinctive flavor and nourishing properties. In our research, we found that lipids like phospholipids, LPLs, and fatty acids were present in relatively low concentrations within chicken soup. The main reason for this is that lipids are insoluble in water, which impedes their efficient release into the chicken soup. Moreover, carbohydrates such as sucrose, trehalose, 1-kestose, maltotriose, L-sorbose, and galactose lactate accumulate in chicken soup, enhancing its sweetness. Furthermore, compared to those in the CM, the relative concentrations of inosine and inosinic acid derived from adenosine and IMP were higher in the CS. These findings suggested that inosine and its derivatives were extracted from the meat and largely dissolved in the soup during the stewing process. Additionally, anserine and carnosine were present in lower concentrations in chicken soup, which helps to reduce its bitter flavor (38). Also, research has shown that prolonging the cooking time facilitates the release of amino acids from the meat into the broth (13). In our study, proline, L-glutamic acid, and L-glutamate were particularly prominent in chicken soup, which was conducive to increasing the sweetness and umami taste of chicken soup. Conversely, the content of Ala, Val, and Tyr was relatively low, which helped to reduce the bitterness and sourness of the chicken soup. In brief, some substances were released from chicken meat into the soup during stewing, contributing to enhancing both the nutritional value and the flavor of chicken soup.

In summary, pretreatment has a negligible impact on the metabolic alterations of chicken meat, but it is highly effective in eliminating the smell of raw meat. Thus, short-heating treatment is also appropriate for lamb and pork processing, allowing for the retention of nutrients while diminishing the smell of raw meat. Meanwhile, stewing, as a mild cooking technique, may also preserve nutrients and enhance the flavor in other meats. Additionally, untargeted metabolomics provided a powerful technique to unveil the overall metabolites profile in black-bone chicken meat and soup during cooking process, which also could be applied to evaluate the nutritional values and flavors under different cooking conditions.

## 4 Conclusion

This study provides insights into alterations in metabolites in Lueyang black-bone chicken meat and soup during stewing. The untargeted metabolomics results demonstrated that short-term pretreatment had little impact on the metabolites of chicken meat. However, stewing had a notable influence on the metabolite profiles of Lueyang black-bone chicken meat. The abundance of partial amino acids, carbohydrates and nucleic acids decreased in chicken meat during the heating process, and these compounds were partially dissolved in the soup. These alterations could be beneficial for reducing bitterness and enhancing sweetness in chicken soup. In addition, the hydrolysis of phospholipids during stewing enhanced the production and accumulation of unsaturated fatty acids and lysophospholipids in fully cooked meat. Further studies are required to explore the flavor compounds and sensory attributes of cooked Lueyang black-bone chicken. Additionally, it is necessary to determine the optimal heating temperature and duration for stewing. In summary, these data provide helpful information for nutritional quality studies on the metabolite profiles of black-bone chicken meat and for the development of high-quality chicken soup products to meet consumer preferences.

## Data Availability

The original contributions presented in the study are included in the article/supplementary material, further inquiries can be directed to the corresponding authors.

## Footnotes

^1^ http://www.hmdb.ca/

^2^ https://metlin.scripps.edu/

^3^ https://cloud.majorbio.com

^4^ http://www.genome.jp/kegg/

^5^ https://docs.scipy.org/doc/scipy/

^6^ https://www.omicshare.com/tools/Home/Soft/getsoft
