# Supplementary material for: Untargeted metabolomics reveals the alteration of metabolites during the stewing process of Lueyang black-bone chicken meat
Source: Front Nutr. 2024 Nov 27;11:1479607. doi: 10.3389/fnut.2024.1479607 (PMC11631612; doi:10.3389/fnut.2024.1479607)
Supplement: Supplementary file 1 [file Image_1.pdf]

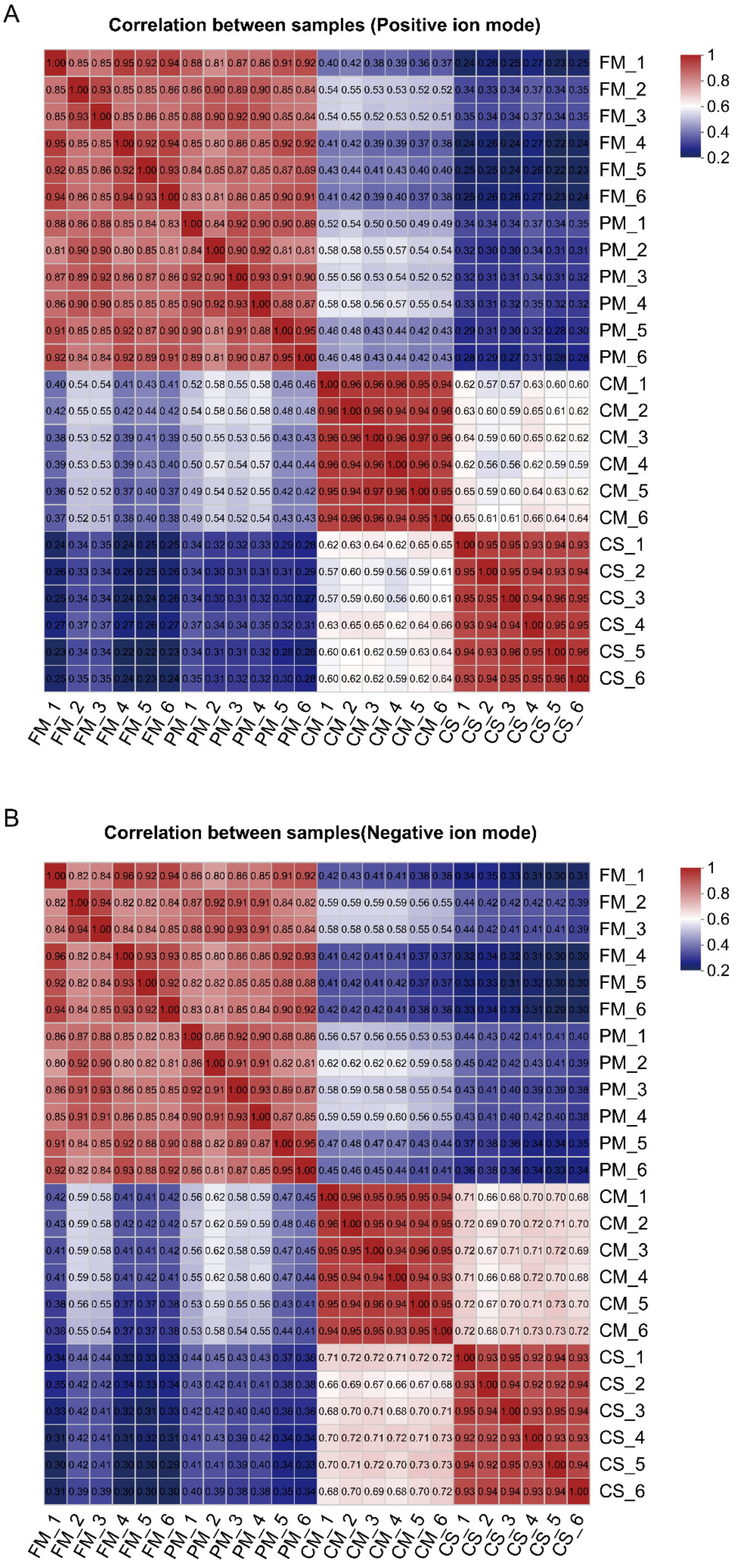

Supplementary Figure 1. Correlation analysis among samples from FM, PM, CM and CS in positive mode (A) and negative mode (B).

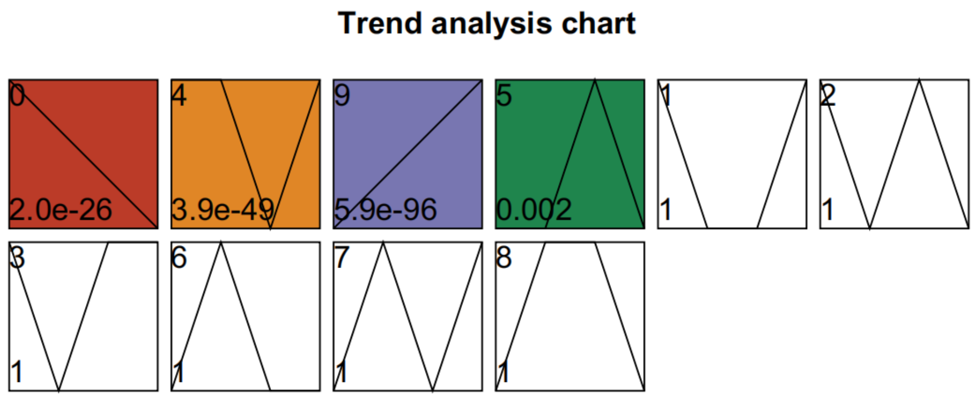

Supplementary Figure 2. Cluster analysis of differential metabolites from FM, PM, CM and CS. The data of cluster 0, cluster 4, cluster 5 and cluster 9 had significant difference.
